# Electron tomography—a tool for ultrastructural 3D visualization in cell biology and histology

**DOI:** 10.1007/s10354-018-0646-y

**Published:** 2018-08-06

**Authors:** Josef Neumüller

**Affiliations:** Center of Anatomy and Cell Biology, Department of Cell and Developmental Biology, Medical University of Vienna, Schwarzspanierstraße 17, 1090 Vienna, Austria

**Keywords:** Electron tomography, 3D visualization, Ultrastructure, Cell biology, Histology, Elektronentomografie, 3D-Darstellung, Ultrastruktur, Zellbiologie, Histologie

## Abstract

Electron tomography (ET) was developed to overcome some of the problems associated reconstructing three-dimensional (3D) images from 2D election microscopy data from ultrathin slices. Virtual sections of semithin sample are obtained by incremental rotation of the target and this information is used to assemble a 3D image. Herein, we provide an instruction to ET including the physical principle, possibilities, and limitations. We review the development of innovative methods and highlight important investigations performed in our department and with our collaborators. ET has opened up the third dimension at the ultrastructural level and represents a milestone in structural molecular biology.

## Zusammenfassung

Die Elektronentomographie (ET) wurde entwickelt, um einige der Probleme zu lösen, die mit der Rekonstruktion dreidimensionaler (3‑D‑)Bilder aus 2‑D-Elektronenmikroskopiedaten von Ultradünnschnitten einhergehen. Durch die stufenweise erfolgende Rotation des Ziels werden virtuelle Schnitte von Semidünnschnitten gewonnen, diese Informationen dienen der Zusammenstellung eines 3‑D-Bilds. Im vorliegenden Beitrag geben die Autoren eine Anleitung für die ET einschließlich der physikalischen Grundlagen, Möglichkeiten und Grenzen. Die Entwicklung innovativer Verfahren wird besprochen, relevante Untersuchungen aus dem Institut der Autoren und in Zusammenarbeit mit anderen werden dargestellt. Durch die ET erschließt sich die dritte Dimension auf der ultrastrukturellen Ebene, sie stellt einen Meilenstein in der strukturellen Molekularbiologie dar.

## Introduction

When inspecting the organization of organelles of a particular cell or the cellular arrangement in several tissues at the ultrastructural level, we usually start from two-dimensional images. We then interpolate a three-dimensional (3D) image from sections at different heights of the same site of an electron microscopic (EM) preparation. The resulting reconstruction, conceived in our imagination, can lead to missing details or general misinterpretations. One of the first successful attempts to overcome these problems concerned the 3D reconstruction from ultrathin serial sections varying between 60 and 80 nm. [1–3]. However, such studies are time consuming and require a skilled technical laboratory staff. Therefore, scientists using electron microscopy sought alternative methods, such as electron tomography (ET).

In this review, we give a short introduction into ET and a historical overview of its development in relevant important centers. We discuss the principle of ET, including possibilities and limitations. We also refer to ET investigations carried out in our department and mention by name scientists who were helpful in establishing this method in our EM laboratory. We also summarize the development of new advanced methods, such as focused ion beam scanning tomography (FIB-SEM), serial block-face scanning electron microscopy (SBF-SEM), axial bright-field scanning transmission electron microscopy (STEM) tomography, and array tomography (AT).

## Technical principles of ET, opportunities and limitations

**Fig. 1:**
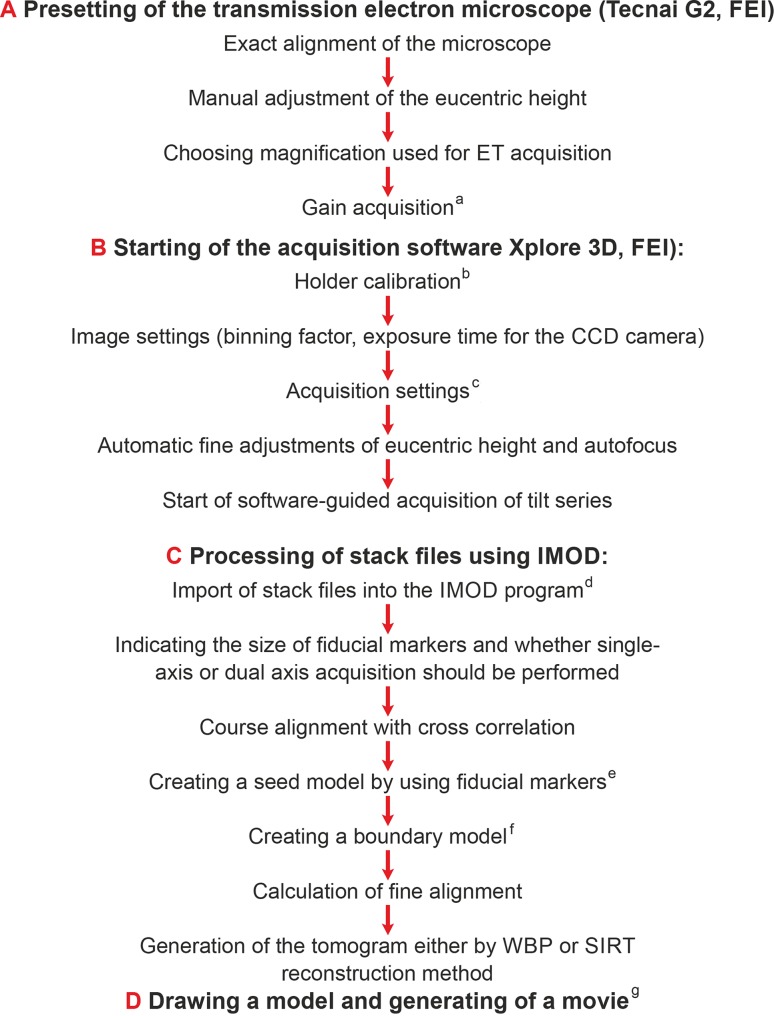
Flowchart showing the sequence of electron tomographic (ET) processing steps as performed with our instrumentation (Tecnai 2G, FEI company, Eindhoven, The Netherlands). Detailed explanations: ^a^Gain acquisition: optimizing the image conditions by performing an illumination intensity calibration curve. ^b^Holder calibration: recording of dislocations of a region of interest (ROI) during acquisition using a particular specimen holder (high-tilt or rotation holder). This setting must be performed only once after changing the cathode (in our case, a LaB_6_ cathode). ^c^Setting of acquisition details: range of tilting angle in the + and − direction, increment for image acquisition, options for fine tuning (tracking before or tracking after taking of an image at a particular tilt angle), choosing whether stack files (*.mrc) or single images should be stored and into which directory. ^d^Import of stack files into the IMOD software (Boulder Laboratories for 3D Electron Microscopy of Cells, University of Colorado, USA): The header of the stack file will read-out by the IMOD software. ^e^Creating a seed model by using fiducial markers: appropriate markers (gold particles) are checked in the horizontal projection and a seed model is generated automatically in which respective markers of every tilt projection are identified. If this procedure doesn’t work automatically because of bad contrast in particular projections, the generation of the seed model can also be performed manually. By running a dual-axis ET, the position of the fiducial markers from the first axis tilt series has to be transferred to the second axis tilt series. ^f^Creating a boundary model allows elimination of image features of no interest by sampling three regions of the tomogram, ones computed from near the top, middle, and bottom of the tilt images. ^g^Drawing a model and creating a movie uses the Amira 5.3.5 software (Mercury Computer Systems, Merignac, Cedex, France). *WBP, SIRT*: reconstruction using Weighted Back Projection or Simulaneous Itinerative Reconstructon Technique respectively

ET takes place in a transmission electron microscope (TEM), in a TEM equipped with scanning device (STEM), or in special scanning electron microscopes at an acceleration voltage of at least 200 kV. In ET using TEM, a semithin section mounted on a grid is inserted into a high-tilt holder, which can be tilted by a eucentric goniometer over a range of maximally −70° to +70°. Typically, depending on the specimen, a range of −65° to +65° is used. The thickness of the semithin sections is restricted to 300 nm in TEM mode and to 1 µm in STEM mode. ET requires a eucentric goniometer with a complex stage, where the grid holder can be moved in directions of the x‑, y‑, and z‑axes. Computer programs for correcting dislocations and providing automatic adjustment of the eucentric height and focus (autofocus) during tilting are therefore indispensable. During tilting, digital images are acquired via a sensitive CCD camera at preset intervals (usually 1°). The tilting limits are set by the edge of the grid and the fixation ring at the holder, which would cover the section at higher tilting angles. This reduces the 3D information and is referred as “missing wedge”. This disadvantage can be partially compensated by carrying out a second tomogram from the same ROI, usually at an angle of 90° to the first axis (dual axis tomography), reducing the missing wedge to a missing cone and providing higher 3D information. The digital images are stored in stack files, which are put through further processing software packages [4]. The next step is the reconstruction, transforming the projections in a Fourier space, from which they are placed as virtual slices into a virtual 3D volume. This method is referred as weighted back projection (WBP). Corresponding structures through the whole 3D space can be traced and connected by the computer in order to obtain a 3D model. Beside the WBP method, the simultaneous iterative reconstruction technique (SIRT; [5]), and the discrete algebraic reconstruction technique (DART; [6]) have been developed in order to improve the image information. Fig. 1 shows a flowchart of the consecutive steps in ET as used in our laboratory. Compared with 3D reconstructions based on serial sections, ET provides significant advantages in z‑axis resolution of the virtual slices which correlates with the voxel size in nanometers, depending on the image resolution and the EM magnification used for ET acquisition.

## Historical overview and state of the art

Investigations using ET started in the last decade of the past century, as summarized in 1997 [7]. Subsequently, several reviews described the technique [8, 9].

To improve the reliability of EM preparations by avoiding chemical artifacts, freezing methods in liquid nitrogen, such as the high-pressure fixation (HPF), were developed. Frozen cells or tissues can be sectioned using cryo-ultramicrotomy. The cryosections can be transferred via a liquid helium-cooled cryostage into a cryo-electron microscope. This method has been applied, above all, by the group of Wolfgang Baumeister at the Department of Molecular Structural Biology, Max Plank Institute for Biochemistry in Martinsried, Germany [7]. Baumeister’s group characterized proteasomes and their subunits by combining crystallographic studies and ET, including single-particle analysis [10]. This group has presented excellent publications on cell organelles [11–15]. Structural and functional cellular aspects were visualized by using special apparative innovations for correlative light and cryo-electron microscopy [16]. An important contribution in neurology concerned the presynaptic organization of vesicles and surrounding filaments [17], as well as the presence of PolyQ inclusions in Huntington disease [18]. Compelling structural research has been done in bacteria [19–21] and viruses [22–25].

The working group of Abraham J. Koster (section Electron Microscopy, department Molecular Cell Biology at the University of Leiden, Netherlands) published articles providing significant technical improvements of ET [26–29]. Several papers contributed to a better understanding of the structure and function of the endoplasmic reticulum, the Golgi apparatus, and related organelles [30–33], as well as of the endothelium-specific Weibel–Palade bodies [34, 35].

Early investigations leading to 3D visualization of the Golgi apparatus were also undertaken by the research group of Kathryn E. Howell at the University of Colorado School of Medicine, Department of Cell and Developmental Biology [36, 37].

## ET investigations carried out in our department

Our primary and central interest was in the 3D visualization of the dynamic structure of the Golgi apparatus in cultured human hepatoblastoma cells under the influence of several environmental factors. ET was implemented in our laboratory in 2004, under helpful instructions by Wim Voorhout (FEI), Bruno M. Humbel, and Jean Luc Murk, Wolfgang Baumeister, Jürgen M. Plitzko, and Matthias Eibauer from the Max Plank Institute of Biochemistry in Martinsried.

In our ET research group of the Department of Cell Biology and Ultrastructure Research (Head: Margit Pavelka), Adi Ellinger was one of the most enthusiastic members together with Margit Pavelka, Josef Neumüller, Claudia Meisslitzer-Ruppitsch, Carmen Ranftler, and Monika Vetterlein, and the technicians Peter Auinger, Ivanna Fedorenko, Ulrich Kaindl, Beatrix Mallinger, Thomas Nardelli, and Elfriede Scherzer.

**Fig. 2:**
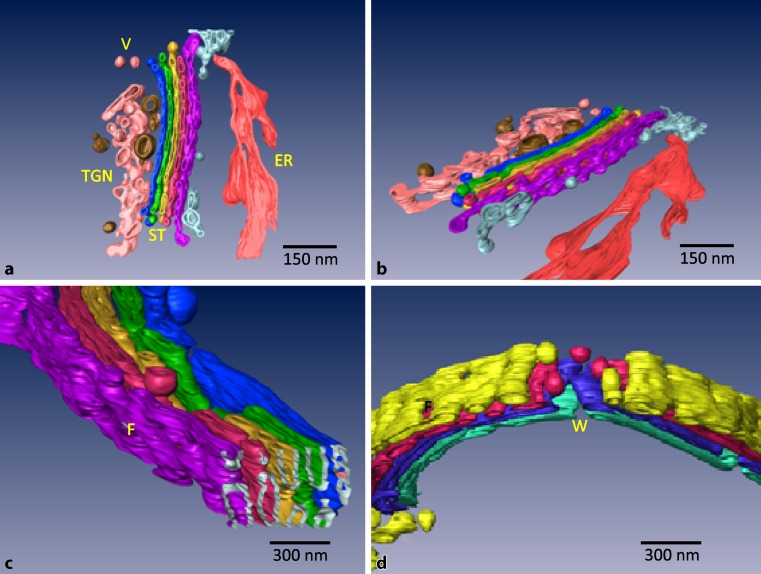
ET model of a Golgi apparatus in HepG2 cells from different views (**a** front view; **b** and **c**: side view). In **a**, the cisternae of the Golgi stack (*ST*), the endoplasmic reticulum (*ER*), vesicular elements (*V*), and the *trans*-Golgi network (*TGN*) are visible. The side views in **b** and **c** show numerous fenestrae at the outmost *cis*- and *trans*-Golgi cisternae. Interruptions of the regular structure of a stack (*W* wells) are shown in (**d**). The colours help to better visualize the details of the different structures. Preparation protocol: high-pressure freezing and cryosubstitution. The models were created by Christoph Weiss

Throughout many years, the complex and dynamic Golgi apparatus architectures were at the center of our interest. To avoid artifacts due to chemical fixation, we used high-pressure freezing followed by freeze substitution and resin embedding (Fig. 2). We visualized the endocytic cellular routes using horseradish peroxidase (HRP)-labeled plant lectin wheatgerm agglutinin (WGA) combined with diaminobenzidine (DAB) cytochemistry [38, 39].

We were interested in correlative microscopy, since light microscopy allows the visualization of structures and metabolic processes using fluorescent markers or tracers while EM can only demonstrate this in fixed, high-pressure cryo-fixed, or frozen specimens but with the advantage of high resolution. Our challenging approach was to combine correlative microscopy with ET: Cell organelles, labeled with fluorescent compounds, were subjected to photooxidation of DAB. In the presence of DAB, cells were photobleached by epifluorescence. Aldehyde-fixed cells on plastic petri dishes were incubated in a DAB solution and irradiated with light at 488 nm, until no fluorescence could be detected. Light microscopically, a dark precipitate was seen at the area of irradiation. This area was marked on the petri dish and further processed for EM. The DAB precipitates at the previously fluorescent sites could then be visualized also at the ultrastructural level [40–43].

The highly dynamic nature of the Golgi apparatus could be studied with the aid of the non-metabolizable glucose analogue 2‑deoxy-D-glucose (2DG). Treatment of cultured cells with 2DG causes dis- and reorganization of the Golgi apparatus stacks and correlated with changes in the cellular ATP levels. The Golgi apparatus is maintained in the form of Golgi bodies, from which the stacks can be rebuilt after 2DG removal [44, 45].

**Fig. 3:**
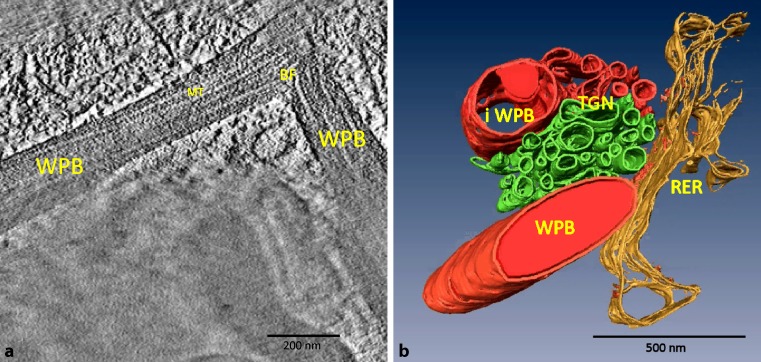
ET of Weibel–Palade (*WPBs*) bodies in human cord blood derived endothelial progenitor cells. In **a**, a bifurcation of a WPB with kinked microtubules (*MT*) is demonstrated in a 2 nm virtual slice. In **b**, an ET model of an immature (*iWPB*) and a mature WPB in connection with RER and TGN is shown. The iWPB is connected to the TGN by a thin bridge where a supply with vWF is hypothesized. Preparation protocol: chemical fixation and embedding in Epon (Agar-100 resin, Agar Scientific Ltd., Stansted, Essex, UK). Modified image version as published in [46]. The model in **b** was created by Jederzeij Kosiuk

Our ET experience in cell biology encouraged other research groups to join and collaborate with us. One project initiated by Johannes Huber, Division of Gynecological Endocrinology and Reproductive Medicine, Medical University of Vienna, dealt with the propagation of endothelial progenitor cells (EPCs) from human cord blood. As a distinct maturation criterion, the formation of endothelium-specific Weibel–Palade bodies (WPBs) was demonstrated. These cigar-shaped rod-like bodies form microtubular structures with a diameter of 13 ± 2 nm. Using ET, we were able to visualize the supply of newly formed WPBs from TGN as well as bifurcated WPBs that could only be demonstrated by ET ([46]; Fig. 3). In cooperation with Herbert Stangl and Clemens Röhrl, Institute of Medical Chemistry, Center for Physiology and Pathophysiology, Medical University of Vienna the uptake and intracellular transport of high-density lipoprotein were followed in EPCs by using fluorescence microscopy and ET [47].

A more recent collaboration involved the research group of José Martínez-Menárguez from the Department of Cell Biology and Histology, Biomedical Research Institute of Murcia (IMIB-Arrixaca-UMU), University of Murcia, Spain. We studied the complex 3D architecture of the Golgi apparatus of principal cells of the rat epididymis [48]. ET revealed an abundant Golgi network and a system of branching interconnected cords.

**Fig. 4:**
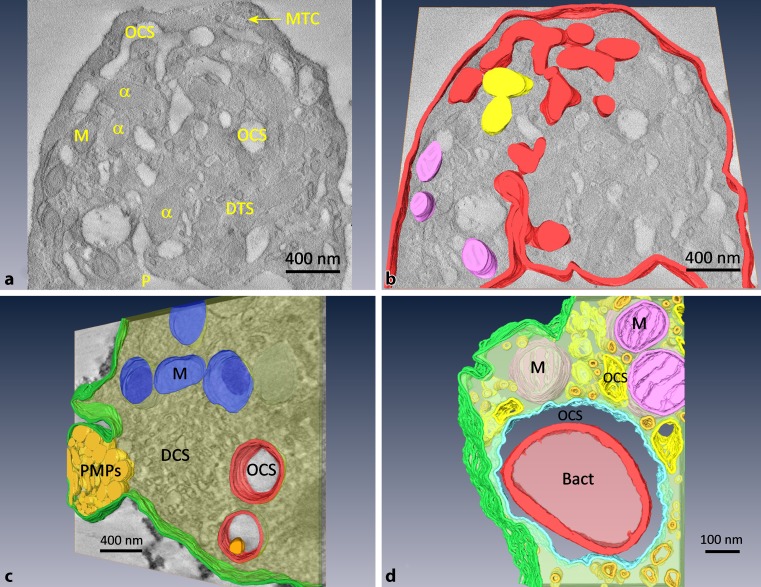
ET of human PLTs. **a** Virtual slice, **b** a model of this. The open canalicular system (OCS) and their pores (*P*) in *red* at the plasma membrane are visible. Alpha granules (*α*) in *yellow*, mitochondria (*M*) in *pink*, the dense tubular system (*DTS*), and the peripheral microtubular ring (*MT*) are visible. **c** ET model of an activated PLT with a protruding sack containing platelet microparticles (*PMPs*) in *yellow* close to the DTS. M are shown in *red*, the OCS in *red*. In **d**, the interaction of a PLT with a *Staphylococcus aureus* bacterium (*Bact*) is visualized in an ET model. The bacterium (in *red*) is completely engulfed by the OCS (in *blue*), as shown by the tracer ruthenium red. Preparation protocol: high pressure freezing and cryosubstitution for **a** and **b** and chemical fixation and embedding in Epon **c** and **d**

A permanent collaboration was set up with the Blood Donation Center of the Austrian Red Cross for Vienna, Lower Austria and Burgenland (Wolfgang R. Mayr, Christof Jungbauer and Renate Renz) and the Department of Blood Group Serology and Transfusion Medicine of the Medical University of Graz (Thomas Wagner). The interaction of platelets (PLTs) with bacteria has been shown in PLT concentrates after spiking with *Staphylococcus aureus*. The uptake of these bacteria into the open canalicular system (OCS) was visualized by using ET [49, 50]. Bacterial contamination leads to activation of PLTs and to the formation of platelet microparticles (PMPs; Fig. 4) responsible for causing unexpected and fatal transfusion reactions [51].

A very challenging project has been carried out together with Robert Borny from the Division of Cardiovascular and Interventional Radiology, Department of Biomedical Imaging of the Medical University of Vienna: ET 3D models were created in order to visualize nucleophilic cross-linked dextran-coated iron nanoparticles [52].

A further collaboration with the research group of Gerd Leitinger and Dagmar Kolb from the Institute of Cell Biology, Histology, and Embryology, Center for Molecular Medicine (ZMM), and the Core Facility “Ultrastructure Analysis”, Center for Medical Research (ZMF), Medical University of Graz focused on investigations of the presynaptic densities in the central nerve system of locusts. Using ET, the architecture of the L‑neuron output synapses revealed that vesicles adjacent to the active zone of the presynaptic density exhibit a highly ordered arrangement, provided by electron-dense filamentous strands. At the site of filamentous extensions of the presynaptic densities, the so called Bruchpilot protein, important for flying and running, was identified by preembedding immunogold labeling [53].

A challenging collaboration started in 2008 with the research group of Kristijan Jezernik Faculty of Medicine, Institute of Cell Biology, University of Ljubljana, Slovenia concerning the nature and function of fusiform vesicles (FVs) in the rat urinary bladder. These organelles represent parts of urothelial plaques, located as microdomains at the apical plasma membrane of the superficial epithelial cells. They are involved in the change of shape of the urothelium during extension or contraction of the urinary bladder. Using ET, we were able to show that these organelles exhibit the shape of flattened discs organized in the form of stacks. It was demonstrated that urothelial plaques contain uroplakins, which are enriched in the post-Golgi compartment [54, 55].

Further ET projects were realized in collaboration with Siegfried Reipert and colleagues from the Core Facility of Cell Imaging and Ultrastructure Research, Faculty of Life Sciences, University of Vienna on the visualization of tubulo-helical membrane arrays (THUMA) in PtK2 rat kangaroo epithelial cell lines [56] and on intracellular mesocrystalloid structures in the ovisac of the sea branchiopod *Artemia franciscana* [57]*.*

### Innovations of the 3D techniques in electron microscopy

In contrast to TEM, which gives insight into cells and tissues by using sections, SEM is a tool to investigate surfaces of specimens. Originally, preparations using a critical point drying apparature followed by palladium-, gold-, or carbon-surface coating could be investigated. A thin electron beam scans the coated surface. Secondary or back-scattered electrons reflected from the coated surfaces are captured by special detectors. However, the disadvantage of coating is the occlusion of fine structures. The introduction of the environment scanning electron microscopes (ESEM) allowed the visualization of nonconductive specimen surfaces without coating. Low-vacuum and low-voltage conditions even allow investigations of living material in a gaseous or humid atmosphere. ESEMs also require special detectors for the respective operating modes. In this respect, scanning transmission electron microscope (STEM) detectors can be used for STEM tomography.

#### Focused ion beam scanning electron tomography

The focused ion beam tomography scanning electron microscope (FIB-SEM) is a special scanning electron microscope equipped with an electron and an additional ion beam (usually of gallium ions). The two beams can be precisely focused on a coincident point of the specimen and the ion beam sputters a small amount of material there. It depends on the force of the current of the ion beam whether the emission of secondary electrons is used for imaging or for cutting away parts of the specimen by milling. As in a conventional scanning electron microscope, the primary beam allows scanning of a part of the surface of the specimen. The possibility of avoiding coating the surface of the specimen in biological samples by charge neutralization using a low energy electron flood gun in order to take a look inside cells and organelles after milling opens up a wide range of applications in life sciences. An encouraging application of FIB-SEM is FIB-SEM tomography, whereby thin sections of down to 3 nm from an ROI of material, embedded in a resin block, are repetitively milled by the ion beam from the surface of the resin block. Images of the newly opened surfaces are acquired and assembled to a 3D volume [58]. New developments have made it possible to thin biological specimens in an FIB-SEM equipped with a cryo-stage. Cryo-samples mounted on grids and thinned by FIB milling can subsequently be processed by using TEM tomography [59, 60].

Another possibility to perform serial sections for tomographic reconstructions lies in the use of serial block-face scanning electron microscopes (SBF-SEM). These instruments contain a built-in ultramicrotome that—similarly to the FIB-SEM—makes it possible to perform serial sections over hundreds of micrometers with excellent resolution [61].

A TEM working in STEM (scanning transmission electron microscopy) mode can be equipped for axial bright-field STEM tomography and works at an acceleration voltage of 300 kV. Since in STEM mode no lenses are present below the specimen, no image blurring owing to inelastic scattering occurs. Dynamic focusing allows a better adjustment of the focus in tilted specimens. Using this method, high-resolution dual axis tilt series from about 1–2 µm thick sections can be performed [62].

#### Array tomography

Array tomography combines fluorescence and electron microscopy. It is appropriate for large-field volumetric imaging of large numbers of antigens, fluorescent proteins, and ultrastructure in individual tissue specimens. Ribbons of ultrathin sections are bonded to a glass slide and stained as desired. Images of the ROIs of every section are acquired, and stack files are aligned and reconstructed to a 3D volume. This method allows performance of correlative microscopy [63, 64].

All these techniques differ significantly in the thickness of the total volume as well as in z‑axis resolution: ET carried out in TEM allows only an investigation of a relatively small thickness of semithin sections of 200–300 nm, but the virtual size differs related to the EM magnification used (for instance 0.39 nm at 29,000x; 0.78 nm at 14,500x; 0.98 nm at 11,500x; acquired with a 4k CCD camera). An equal resolution can be achieved by material milling in a cryo-FIB-SEM with subsequent transfer of the specimen to TEM for ET [60]. Using axial bright-field STEM-ET, the thickness of the semithin section can be extended to 1–2 µm, but the thickness of the virtual slices decreases to 3–5 nm [62]. SBF-SEM allows a 3D reconstruction of volumes up to 600 nm, but the thickness of the slices remains—depending on the material and the resin of the block—in the range of 25–50 nm [65]. Therefore, these techniques complement each other and should be applied according to the respective issue.

These are only the beginnings of a methodology that will increase our knowledge of functional histology and cell biology by bridging the imaging possibilities at the LM and EM level. Therefore, the access to the third dimension at the ultrastructural level was a milestone in life sciences and helpful for developing a better understanding of structural molecular biology.
